# Direct Screw Osteosynthesis for an Elderly Osteoporotic Patient With C2 Complex Fracture

**DOI:** 10.7759/cureus.42510

**Published:** 2023-07-26

**Authors:** Yoshitaka Nagashima, Yusuke Nishimura, Mamoru Matsuo, Takafumi Tanei, Ryuta Saito

**Affiliations:** 1 Neurosurgery, Nagoya University Graduate School of Medicine, Nagoya, JPN

**Keywords:** osteoporosis, osteosynthesis, atypical c2 fracture, c2 complex fracture, c2 fracture

## Abstract

There are various types of C2 fractures, including odontoid fractures, hangman fractures, and complex fractures, which involve the vertebral body or multiple fracture types. The published literature on C2 complex fractures is limited, and treatment strategies have not yet been established.

An 80-year-old woman with a history of osteoporosis, brain stroke, and cervical spondylosis fell and sustained a C2 complex fracture. Initial treatment with a cervical collar was unsuccessful and a C2 direct screw osteosynthesis surgery was performed under an image-guided three-dimensional navigation system. The surgical procedure was successfully performed with a surgical time of 83 minutes and a blood loss of 31 ml. Her neck pain improved after surgery. Follow-up CT scans revealed acceptable healing of the fracture four months later.

C2 direct screw osteosynthesis is a viable treatment option for C2 complex fractures, particularly in elderly patients who may benefit from early stabilization of the fracture to prevent complications associated with long-term conservative treatment.

## Introduction

C2 fractures are frequently caused by traffic accidents or falls from height [1,2]. In elderly individuals, falls from a standing position are known to be the most prevalent cause, accounting for 47% of all C2 fractures [3]. Among C2 fractures, odontoid fractures and hangman fractures are the most frequent and well-documented fracture types. C2 complex fractures, excluding the above, are characterized by the inclusion of a vertebral body or multiple fracture types. C2 complex fractures, distinct from the aforementioned types, are characterized by the involvement of the vertebral body or the presence of multiple fracture types.

The literature on C2 complex fractures is limited and the majority of studies focus on odontoid fractures and hangman fractures [2]. Treatment options for C2 complex fractures include conservative treatments, such as the use of cervical collars, and surgical treatments, such as direct screw osteosynthesis. Despite the primary objective of these treatments being spinal stabilization and the prevention of neurological deterioration, a standardized treatment protocol is yet to be established.

Surgical treatment is often deemed unsuitable in the elderly due to systemic complications, such as cardiac and pulmonary disease. Conversely, there are also significant problems with conservative treatment, such as impaired bone healing due to osteoporosis, non-compliance to treatment protocols, and diminished activities of daily living (ADL) resulting from extended bed rest. However, recent developments in minimally invasive surgery and navigation technology have made surgery safer and more feasible. In this case report, we present a case that offers valuable insights into the intricacies of managing C2 complex fractures in the elderly. We further explore the potential advantages of direct screw osteosynthesis as a viable treatment option.

## Case presentation

**Figure 1:**
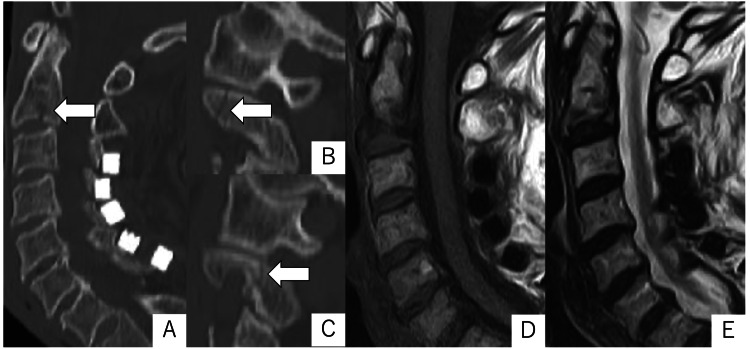
Sagittal CT images (A: midline, B: right, C: left) and magnetic resonance (MR) images (D: T1W, E: T2W) obtained in the early days after the injury. CT images showed fracture lines in the C2 pedicle and vertebral body (arrows). MR images showed signal changes in the vertebral body, but no evidence of disc or ligament injury. T1W: T1-weighted; T2W: T2-weighted.

An 80-year-old woman, receiving treatment for osteoporosis, presented with severe neck pain after a ground-level fall at her residence. She had exhibited right incomplete hemiparesis, aphasia, and executive function disorder due to a history of brain stroke. She had also previously undergone laminoplasty for cervical spondylosis. Computed tomography (CT) imaging of the cervical spine demonstrated C2 pars and pedicle fractures and vertebral body fractures without dislocation (Figure 1). Magnetic resonance imaging (MRI) showed no evidence of intervertebral disc or ligamentous injuries (Figure 1). Based on these imaging findings, the patient was made a diagnosis of osseous C2 complex fracture.

**Figure 2:**
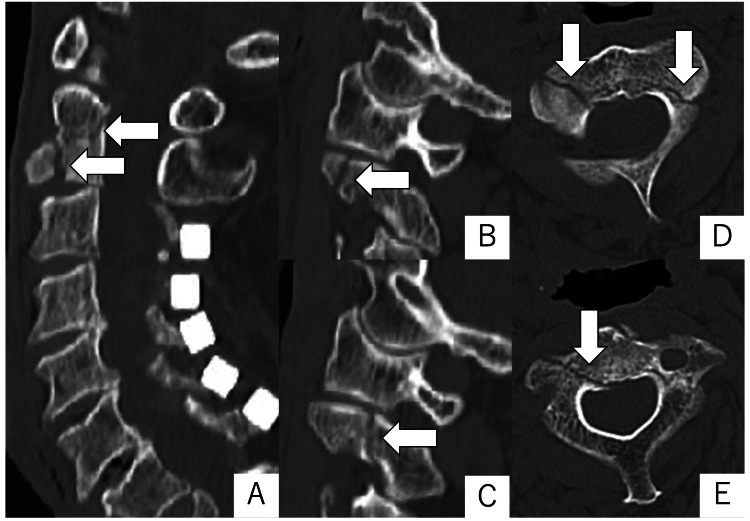
CT images two weeks after the injury. CT imaging two weeks after the injury revealed an enlarged fracture line and dislocation in progression (arrows). A-C: sagittal; A: midline; B: right; C: left; D, E: axial.

Considering the patient's overall health status, conservative treatment with a rigid cervical collar was chosen over a halo vest. However, she could not tolerate the rigid cervical collar due to her executive dysfunction. Two weeks later, a CT scan revealed displacement of the C2 fracture line (Figure 2), prompting us to proceed with the surgical procedure.

**Figure 3:**
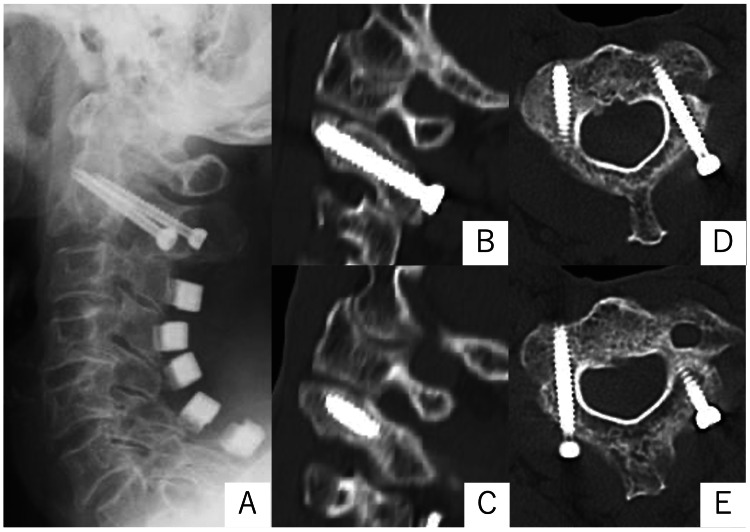
Radiographic images after surgery. X-ray obtained immediately after surgery shows that the screws are in place without any problems. (A) CT images four months later already confirmed acceptable fracture healing. B, C: sagittal; B: right; C: left; D, E: axial.

Under general anesthesia, the patient was placed in a prone position with a head pin fixation, and motor-evoked potential (MEP) recordings were performed. A midline skin incision was made posterior to the neck. The paraspinal muscle was dissected and the C2 spinous process, C2 laminae, and the entry point of the screw were exposed. A reference arm of the navigation was fixed on the C2 spinous process. Under the three-dimensional navigation guide, the pilot hole was created by a navigation-guided drill to avoid excessive force on affected fractures. The drill hole passed through the fracture site. Then bilateral cannulated C2 lag screws were inserted into the C2 laminae penetrating through the pars fracture lines. The surgery was successfully completed with a surgical time of 83 minutes and a blood loss of 31 ml. The displaced bilateral pars interarticularis were successfully repositioned and stabilized. Her neck pain dramatically improved after surgery. Follow-up cervical CT scans at four-month postoperatively revealed acceptable fracture healing (Figure 3).

## Discussion

C2 complex fractures may have a variety of treatment options depending on the state of injury and the patient's systemic condition. The case presented here provides valuable insight into the management of complex C2 fractures and focuses on direct screw osteosynthesis as a viable treatment option. This technique is a minimally invasive technique aiming to preserve the range of motion of affected spinal segments. This technique is usually indicated for pure osseous injuries without disc injuries, ligamentous injuries, or dislocations. This procedure has been reported in numerous articles as a treatment for selected C2 hangman's fractures [4,5] and has also been used in the treatment of C2 complex fractures with favorable outcomes [6,7].

C2 complex fractures are characterized by the presence of C2 body involvement or multiple fracture types except for pure odontoid or hangman's fractures. Benzel et al. classified fractures involving the C2 body as coronally oriented type, sagittally oriented type, and horizontal rostral type [8]. The horizontal rostral type is the same as the so-called type III odontoid process fracture described by Anderson and D'Alonzo. In patients without obvious instability, most fractures have been treated conservatively, though the clinical results have not always been satisfactory [7,9]. Therefore, some patients require spinal fixation. Direct screw osteosynthesis is suitable for only a coronally oriented type, also known as an atypical traumatic spondylolisthesis or unusual type of hangman’s fracture [9]. The present case belongs to the coronally oriented type and requires appropriate management due to the potential for delayed neurological damage if the bony fusion failure occurs.

The safety of the surgical procedure has been improved by recent developments in navigation surgery [10,11]. The risk of injury to the vertebral artery and other vital structures of concern in direct screw osteosynthesis procedure was minimized by an image-guided three-dimensional navigation system [10]. However, even if navigation is used, other techniques must be chosen instead of osteosynthesis if anatomical abnormalities, including high-riding vertebral artery or narrow C2 pedicle, are present. Also, a C2 direct screw osteosynthesis is not possible if the lateral mass is comminuted. Nevertheless, this technique should be considered first in surgical treatment due to its advantage in preserving the range of motion of the joint. The case we presented highlights the potential for technological advances to improve the safety and feasibility of surgical intervention in high-risk populations. In other words, the procedure may be acceptable to employ at an early stage in patients who are expected to be difficult to treat conservatively and have a low success rate of bony fusion.

The use of direct screw osteosynthesis in our patient resulted in immediate stabilization of the fracture and significant improvement in neck pain, allowing early mobilization. This result is particularly important in the elderly population, where long-term immobilization may lead to a reduction in overall health and quality of life. Immediate stabilization of the fracture via direct screw osteosynthesis can also prevent complications associated with long-term immobilization via a halo vest or rigid cervical collar. Although surgical treatment is generally hesitated in the elderly, the benefits of the C2 direct screw osteosynthesis in older patients are significant. However, it is important to note that the decision to undertake surgical intervention should always be made on a case-by-case basis, taking into account the overall health of the patient, the nature of the fracture, and the potential risks and benefits of surgery. In addition, with future developments in conservative treatment, including medication, and advances in surgical techniques, optimal treatment will continue to evolve. Although the present case report suggests the potential benefits of direct screw osteosynthesis, further research is needed to establish a standardized treatment protocol for C2 complex fractures.

## Conclusions

C2 osteosynthesis, one of the preferred treatment options for C2 complex fractures, has many advantages, especially for elderly patients who have difficulty with conservative treatment.

## References

[1] Turtle J, Kantor A, Spina NT, France JC, Lawrence BD (2020). Hangman's fracture. Clin Spine Surg.

[2] Tadros A, Sharon M, Craig K, Krantz W (2019). Characteristics and management of emergency department patients presenting with C2 cervical spine fractures. Biomed Res Int.

[3] Radovanovic I, Urquhart JC, Rasoulinejad P, Gurr KR, Siddiqi F, Bailey CS (2017). Patterns of C-2 fracture in the elderly: comparison of etiology, treatment, and mortality among specific fracture types. J Neurosurg Spine.

[4] ElMiligui Y, Koptan W, Emran I (2010). Transpedicular screw fixation for type II hangman's fracture: a motion preserving procedure. Eur Spine J.

[5] Schleicher P, Scholz M, Pingel A, Kandziora F (2015). Traumatic spondylolisthesis of the axis vertebra in adults. Global Spine J.

[6] Rehman RU, Akhtar MS, Bibi A (2022). Case of pedicle lag screw fixation for oblique axis body and pars fractures with displacement. Surg Neurol Int.

[7] Reynolds JA, MacDonald JD (2016). Direct C2 pedicle screw fixation for axis body fracture. World Neurosurg.

[8] Benzel EC, Hart BL, Ball PA, Baldwin NG, Orrison WW, Espinosa M (1994). Fractures of the C-2 vertebral body. J Neurosurg.

[9] Zhang YS, Zhang JX, Yang QG, Shen CL, Li W, Yin ZS (2014). Surgical management of the fractures of axis body: indications and surgical strategy. Eur Spine J.

[10] Singh PK, Verma SK, Garg M (2017). Evaluation of correction of radiologic parameters (angulation and displacement) and accuracy of C2 pedicle screw placement in unstable hangman’s fracture with intraoperative computed tomography-based navigation. World Neurosurg.

[11] Nagashima Y, Nishimura Y, Haimoto S (2021). Piecemeal resection of aggressive vertebral hemangioma using real-time navigation-guided drilling technique. Nagoya J Med Sci.

